# Pre-orthodontic Orofacial Myofunctional Therapy With Myosimple: A Case Report

**DOI:** 10.7759/cureus.95279

**Published:** 2025-10-23

**Authors:** Philippe Amat

**Affiliations:** 1 Orthodontics and Dentofacial Orthopaedics, Private Practice, Le Mans, FRA

**Keywords:** orofacial disorders, orofacial myofunctional therapy, orthodontics, orthodontic treatment time, prefabricated functional appliance

## Abstract

This case report describes how to begin multi-attachment or aligner orthodontic treatment with a phase of orofacial myofunctional therapy (OMT) combined with the use of Myosimple, a new prefabricated functional appliance (PFA). An 11-year-old patient presented with a Class I malocclusion tending towards Class II Division 2, 100% incisor overbite, maxillary and mandibular crowding, and atypical lateral swallowing. Treatment began with an OMT phase combined with the use of a Myosimple, which was worn for an average of 13-14 hours per day during the week, i.e. during sleep and during the day outside of school, with more regular wear at weekends. The Myosimple was immediately well accepted and worn thanks to its innovative design and very small size. Within three months, wearing the Myosimple corrected the incisor overbite, improved the coordination of the dental arches, reduced maxillary and mandibular crowding, and enabled functional swallowing. This improvement in the shape and relationship of the dental arches then allowed for more effective and faster continuation of treatment with multi-attachments or aligners.

## Introduction

Orofacial myofunctional disorders (OMDs) are abnormal muscle movements resulting from structural differences and involving complex interactions between orofacial structures and their daily functions. The main ones are oral breathing, atypical anterior or lateral swallowing, abnormal labial resting posture, lip incompetence, thumb sucking and general sucking habits, masticatory dysfunction, and bruxism [1]. Orofacial myofunctional therapy (OMT) is the rehabilitation of the muscles, functions, and resting postures of the orofacial complex. It mainly involves isotonic and isometric exercises which, by targeting the oral and oropharyngeal structures, are combined with specific breathing, swallowing, chewing, and posture exercises. OMT is used in the therapeutic management of orofacial dysfunctions in patients of all ages with a wide range of disorders and comorbidities [2,3].

There appears to be a link between different types of malocclusions and orofacial dysfunctions [4,5]. The morphogenetic role of the tongue mainly occurs at rest and during breathing in three dimensions. Oral breathing is associated with many craniofacial dysmorphisms. Regarding swallowing, phonation, non-nutritive sucking, and temporomandibular joint dysfunction, it is the combination of several anomalies that is found in dysmorphic disorders, without a cause-and-effect relationship having been clearly established [5]. It is essential to understand the underlying pathologies contributing to malocclusions. OMT is a key factor in achieving better functional orofacial balance and contributing to the stability of orthodontic treatments. A recent systematic review of the literature [6] concluded, based on 14 published studies (1,105 subjects studied) with heterogeneous levels of bias, that OMT combined with the use of a prefabricated functional appliance (PFA) was superior to implement OMT without PFA. When the superiority of a treatment has been demonstrated, it is preferable that clinicians use it, all other factors and conditions being equal [7]. This appears to be the case with PFA-assisted OMT. This approach appears to be a necessary paradigm shift that would be useful to make available to our patients. After more than 20 years of experimentation, analysis of published data and discussions with numerous orthodontists, physiotherapists, and speech therapists, we have designed and patented a new PFA. It differs from other PFAs in several innovative features, such as its specific tongue ramp (TR), which promotes tongue propulsion and frees the upper airways. The rigidity of the material used to make Myosimple prevents it from deteriorating while maintaining excellent comfort thanks to its very small size. It may seem paradoxical, but patients accept it immediately, feeling more stable and comfortable than with a soft PFA.

## Case presentation

**Figure 1:**
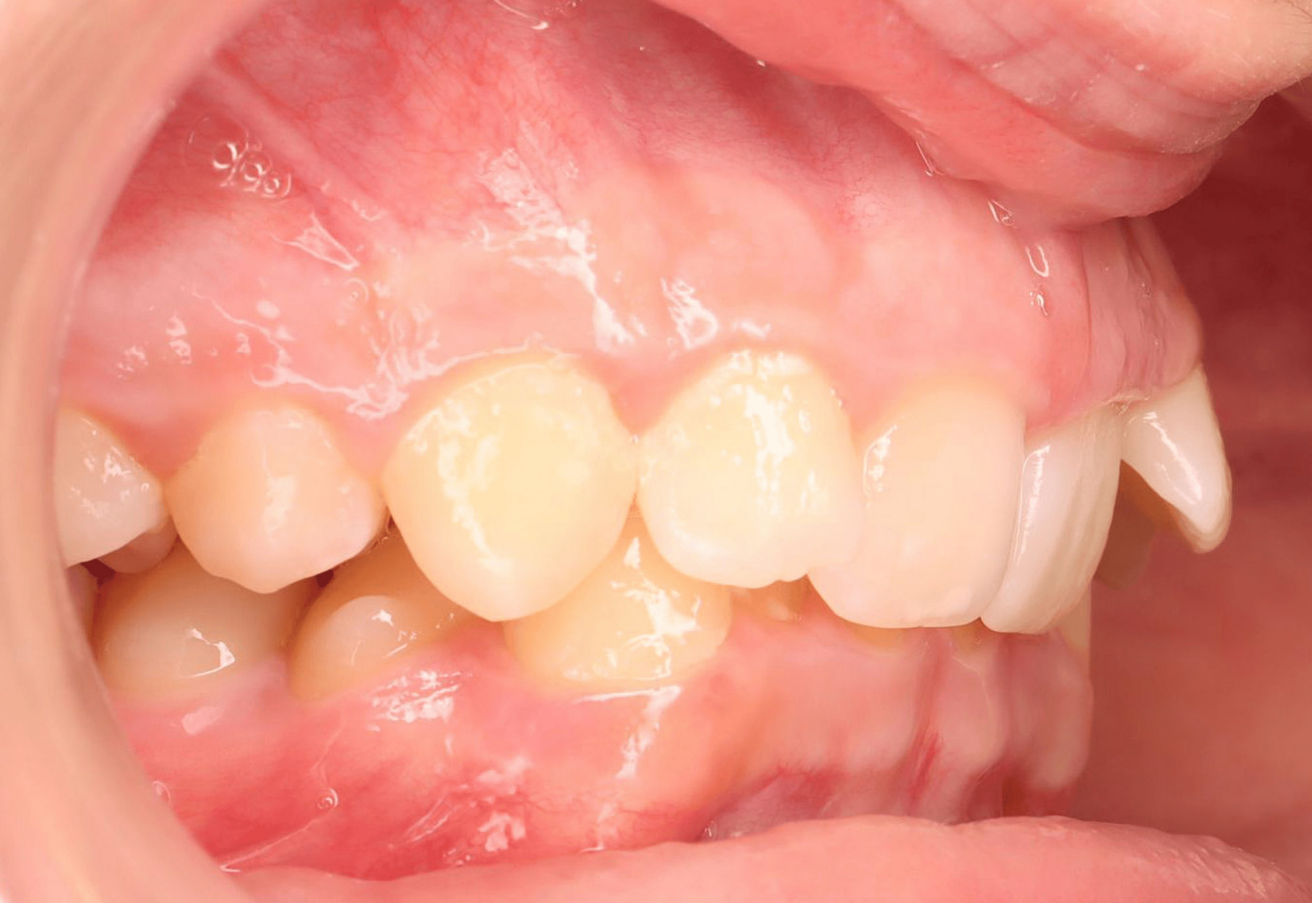
Pre-treatment intraoral photographs (right view).

**Figure 2:**
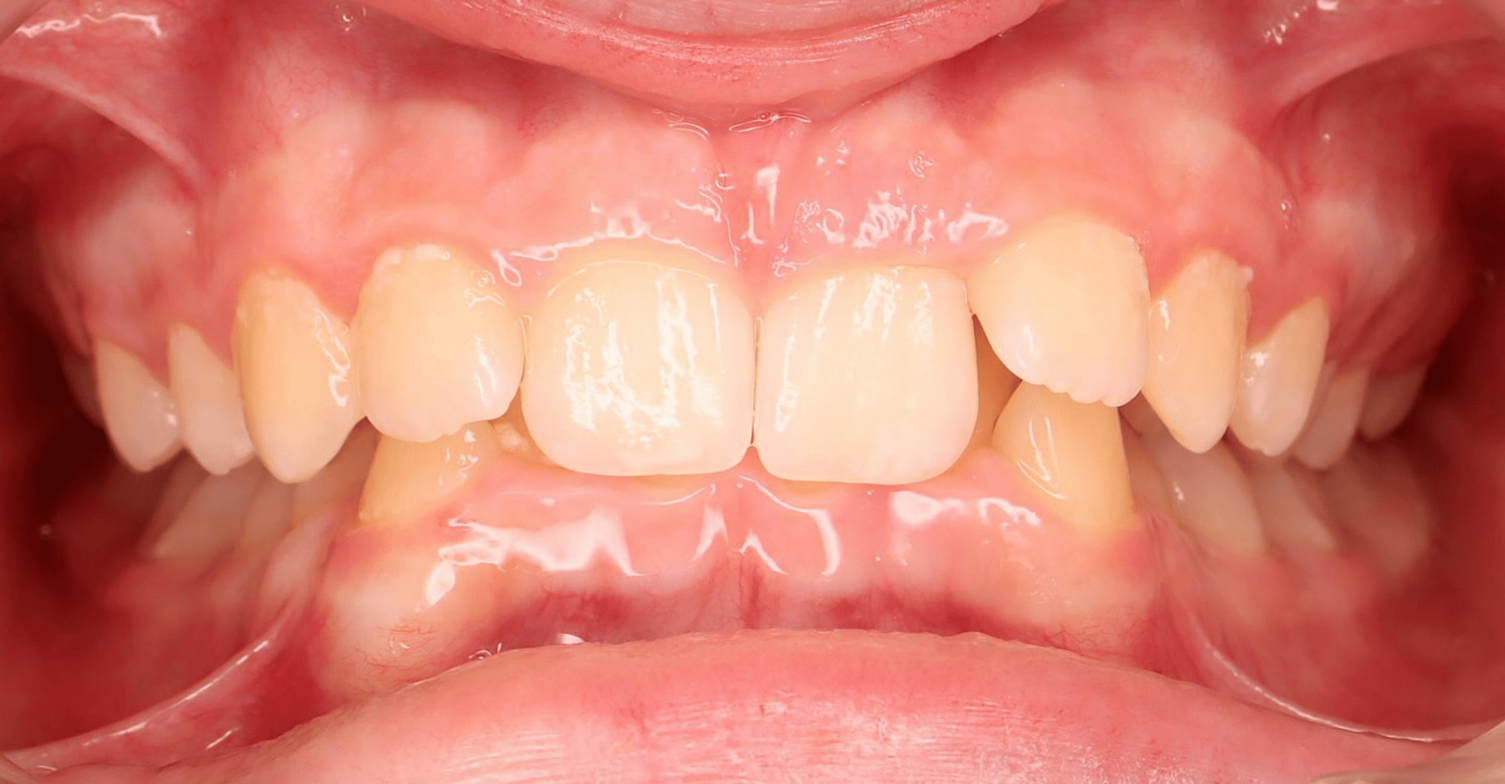
Pre-treatment intraoral photographs (frontal view).

**Figure 3:**
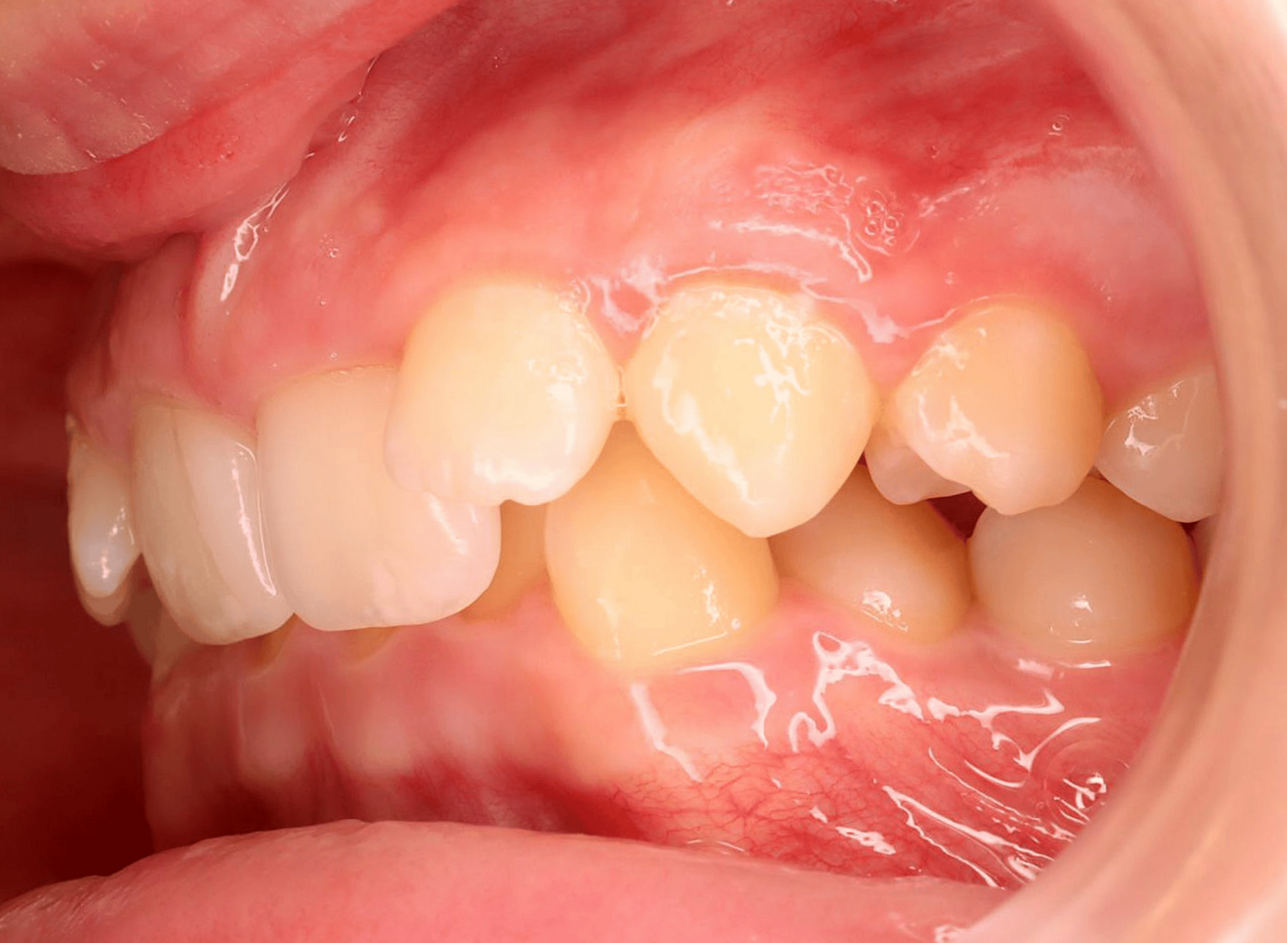
Pre-treatment intraoral photographs (left view).

**Figure 4:**
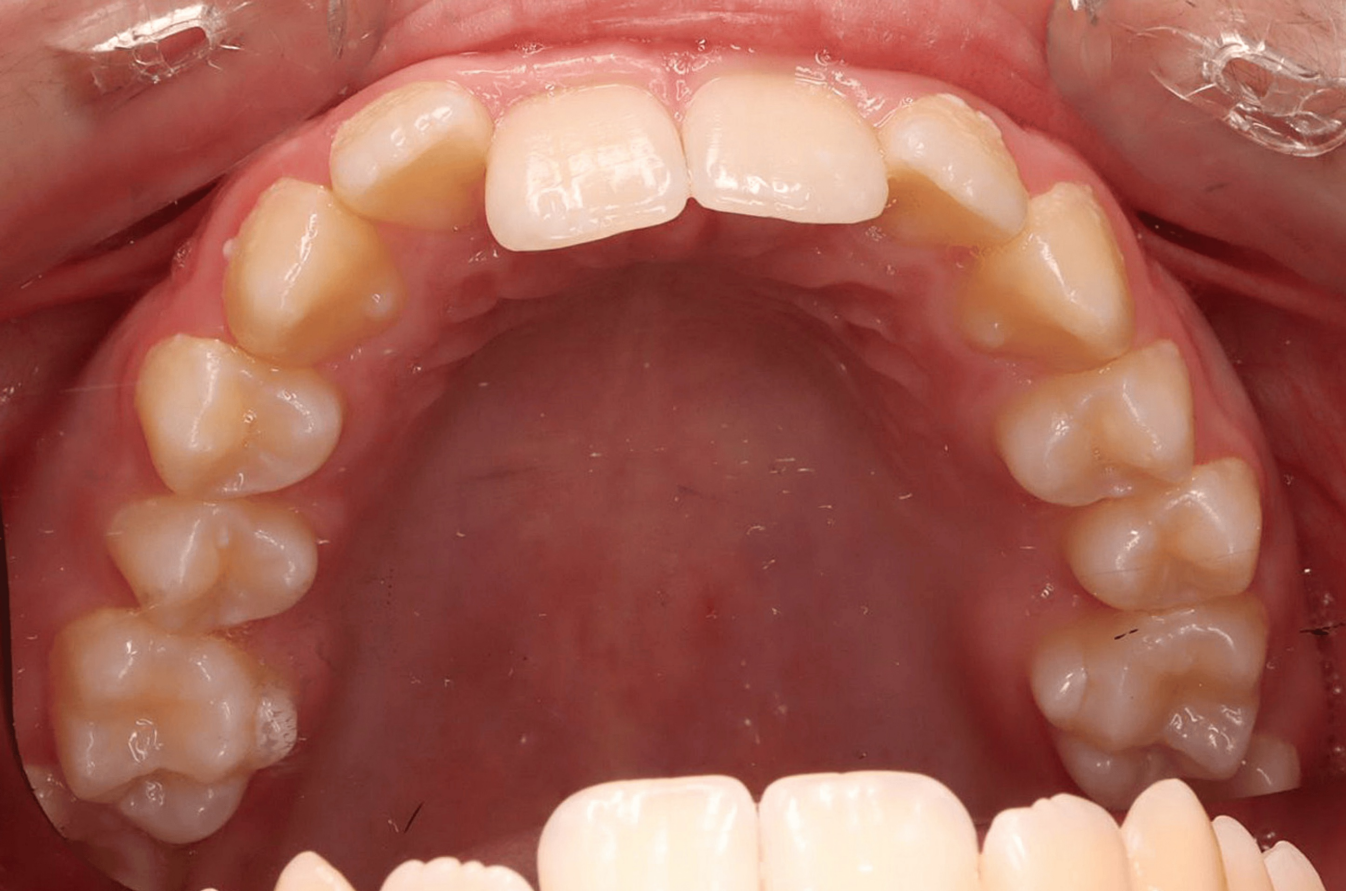
Pre-treatment intraoral photographs (maxilla occlusal view).

**Figure 5:**
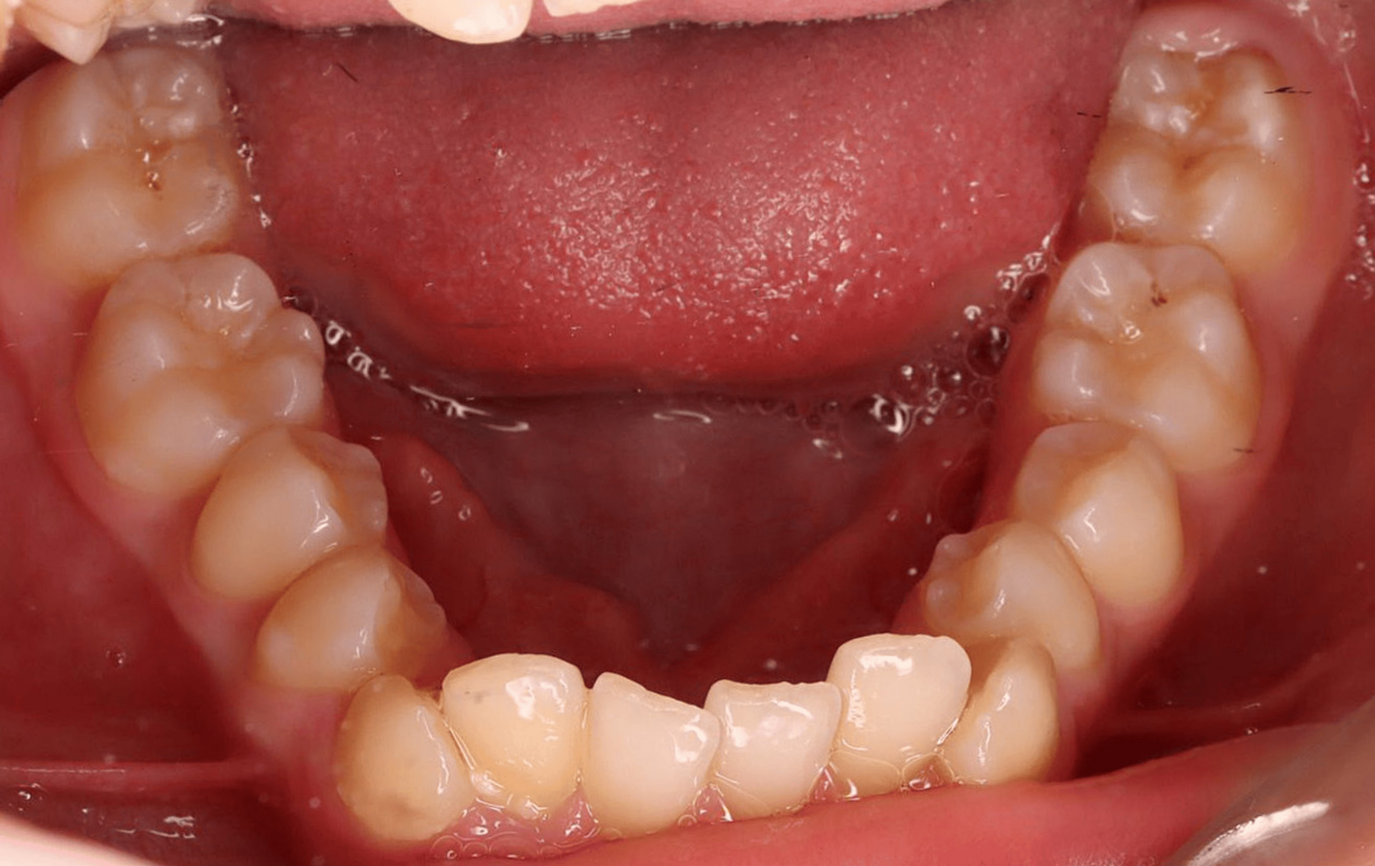
Pre-treatment intraoral photographs (mandibular occlusal view).

A young patient, aged 11 years and 8 months, presented with Class I malocclusion tending towards Class II Division 2 with a 3 mm incisal overjet, 100% incisal overbite, maxillary and mandibular crowding, and vestibular ectopias of the right maxillary second premolar and left maxillary first premolar (Figures 1-5). During the closure phase, there was no premature contact with the lingualized maxillary incisors. The patient was at the beginning of his adolescent growth spurt.

**Figure 6:**
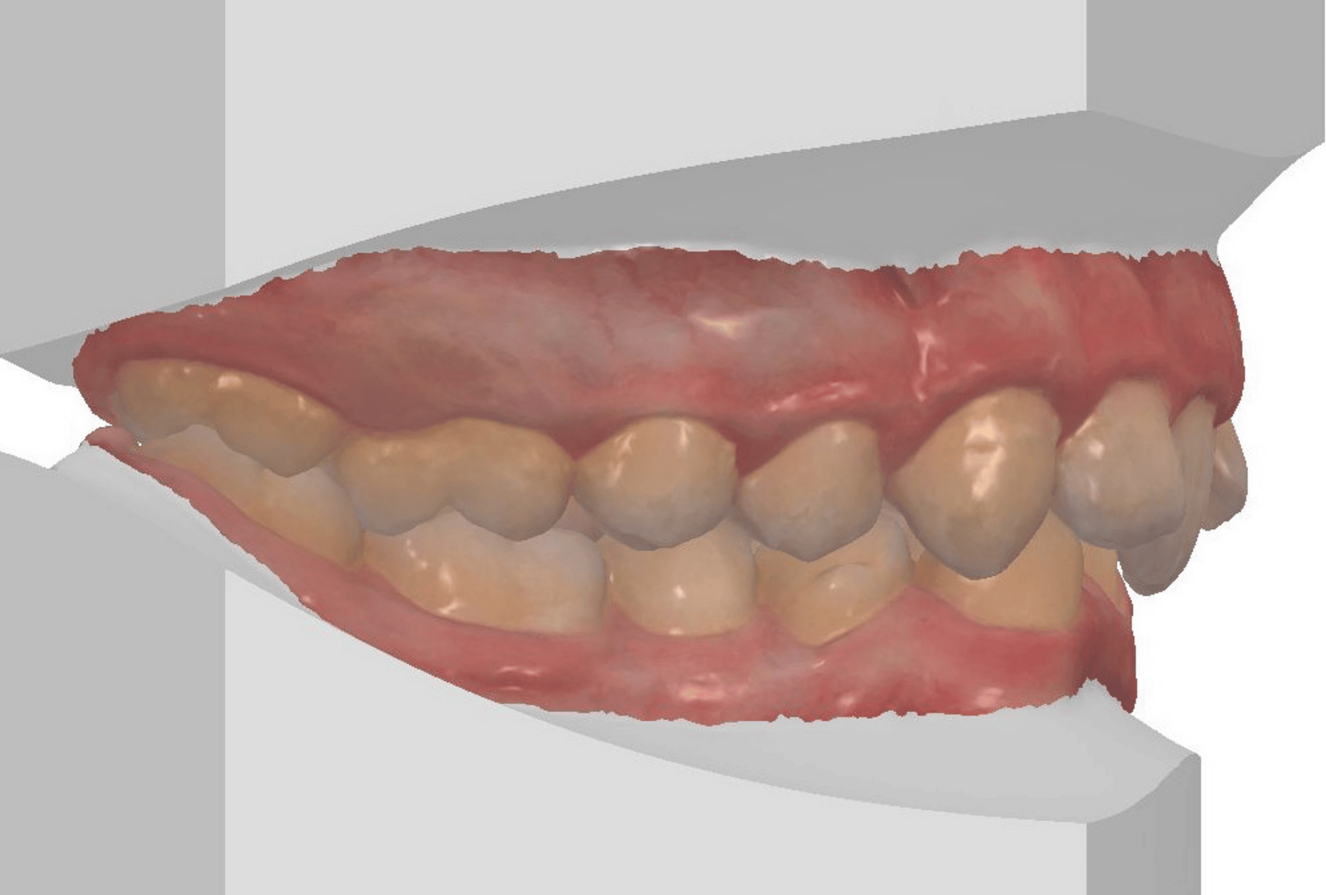
Pre-treatment 3D digital impression (right view).

**Figure 7:**
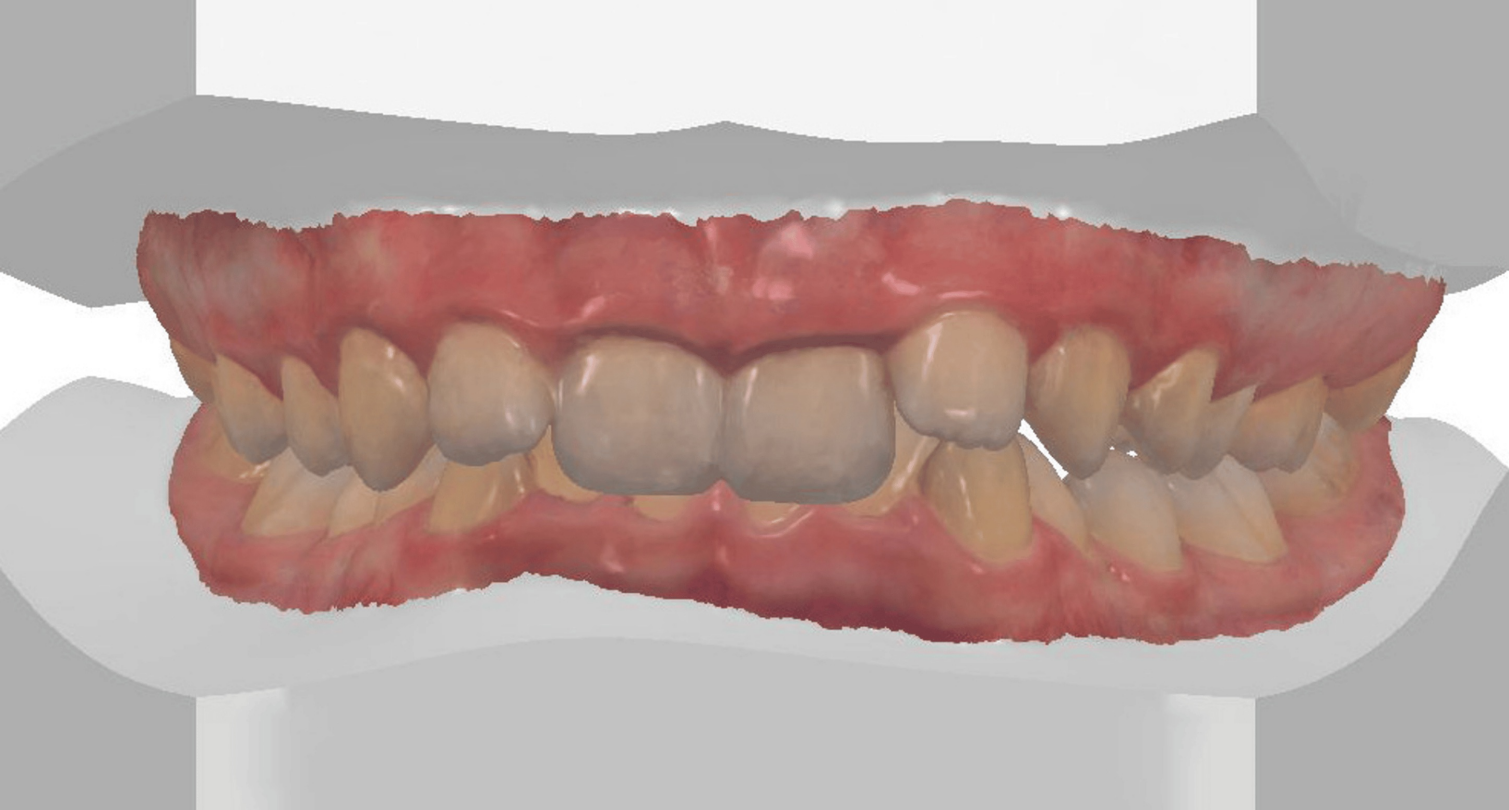
Pre-treatment 3D digital impression (frontal view).

**Figure 8:**
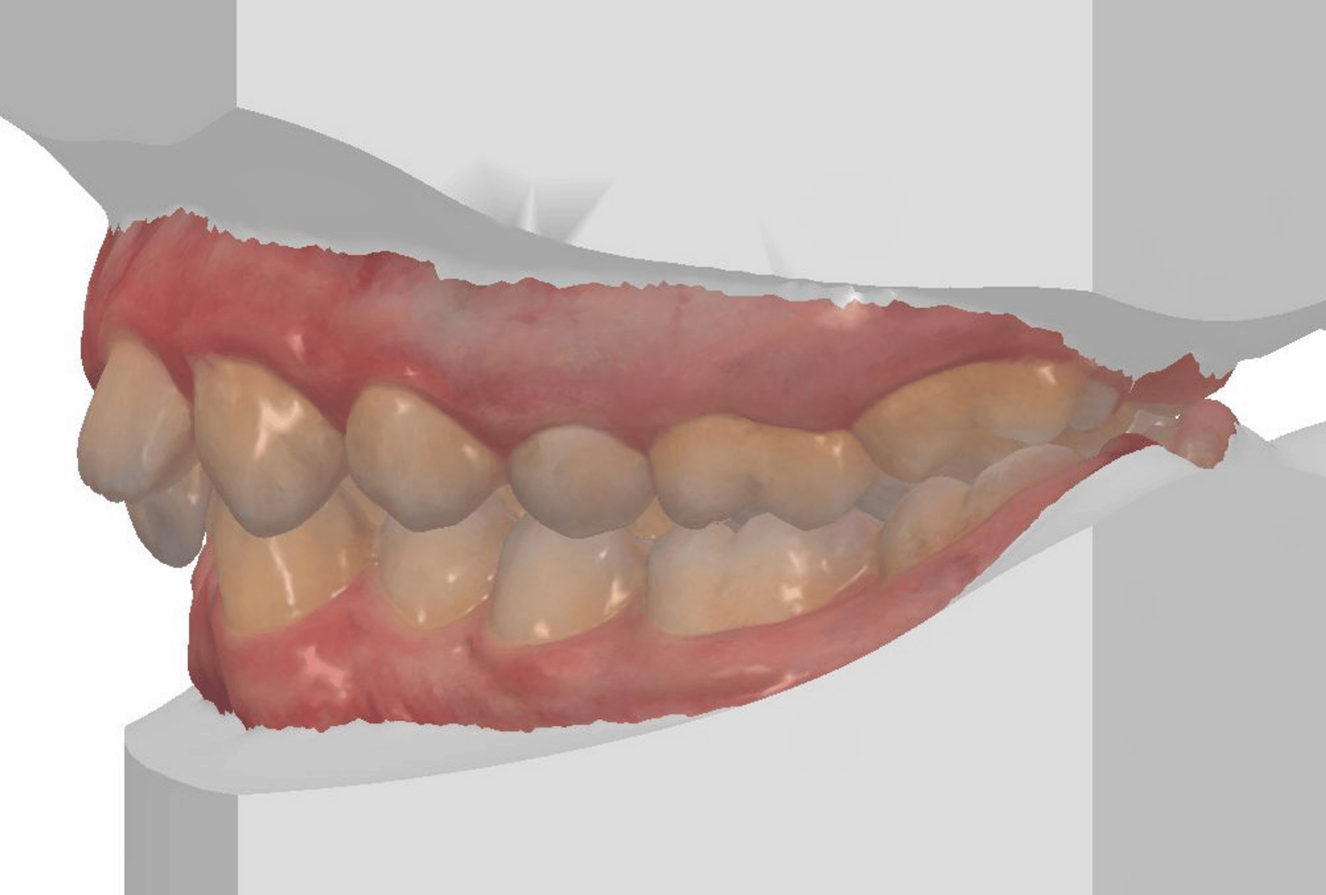
Pre-treatment 3D digital impression (left view).

The 3D digital impression views allow for an assessment of molar relationships that are not visible on intraoral photographs taken prior to treatment (Figures 6-8).

**Figure 9:**
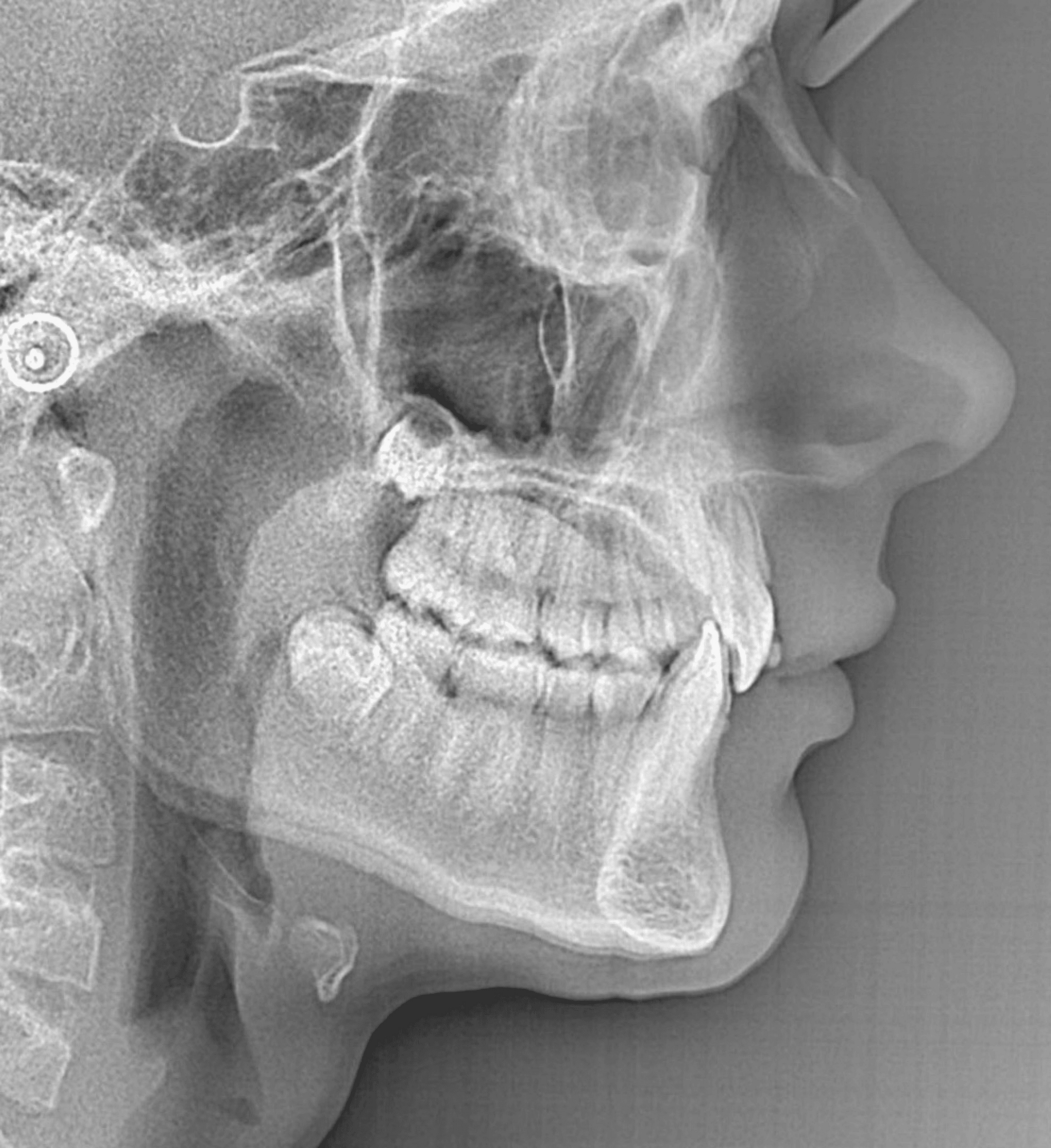
Pre-treatment profile teleradiograph.

His ventilation was nasal, and he presented with atypical swallowing with lateral interposition of the tongue. This interposition in the premolar-molar areas was revealed during the clinical examination. Tongue volume and mobility were normal. The profile teleradiograph showed the absence of tonsils and adenoids (Figure 9).

**Figure 10:**
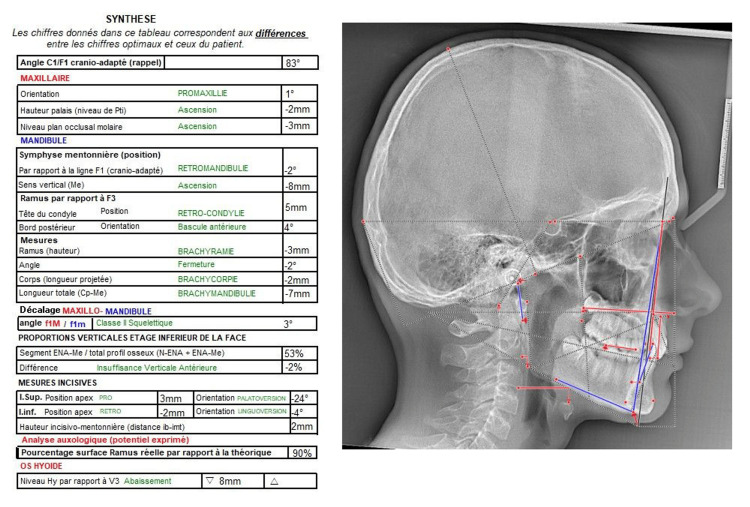
Prof. Jean Delaire's cephalometric analysis of the pre-treatment profile teleradiograph.

Prof. Jean Delaire's cephalometric analysis of the pre-treatment profile teleradiograph showed a slight skeletal Class II malocclusion with anterior vertical deficiency, a 24° lingual version of the maxillary central incisors, and a 4° lingual version of the mandibular incisors (Figure 10).

Treatment began with a phase of OMT, assisted by the wearing of a rigid PFA. The lingual version of the maxillary central incisors could have been corrected by other devices such as a quad-helix combined with incisor brackets. Treatment could also have been started directly using aligners. The decision was made to use Myosimple to both improve anterior guidance and assist in the rehabilitation of dysfunctional swallowing.

**Figure 11:**
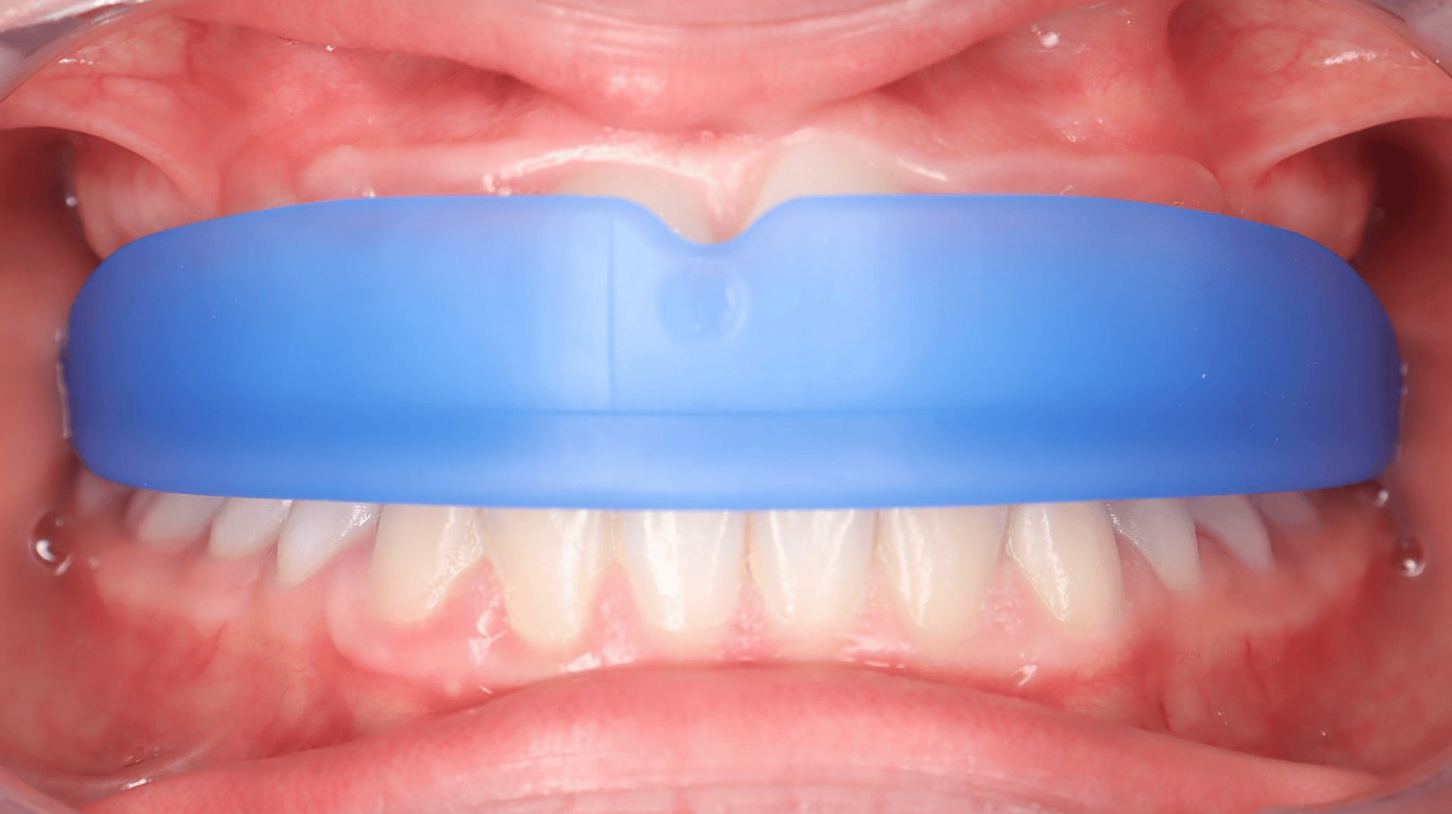
The Myosimple, a new PFA. PFA: prefabricated functional appliance.

The PFA was a Myosimple (Figure 11), a new innovative appliance that made it possible to correct the incisive overbite in three months with an average daily wear time of 13-14 hours per week, during sleep and during the day outside of school, with more consistent wear on weekends.

**Figure 12:**
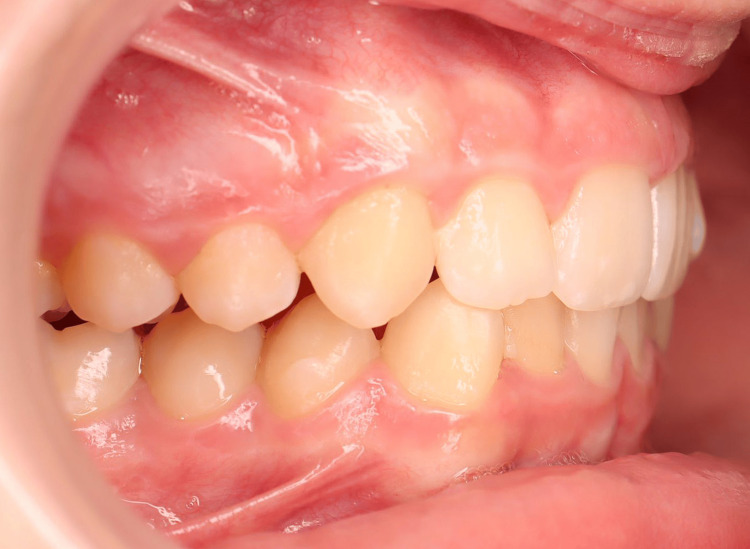
Intraoral photographs after wearing Myosimple for three months (right view).

**Figure 13:**
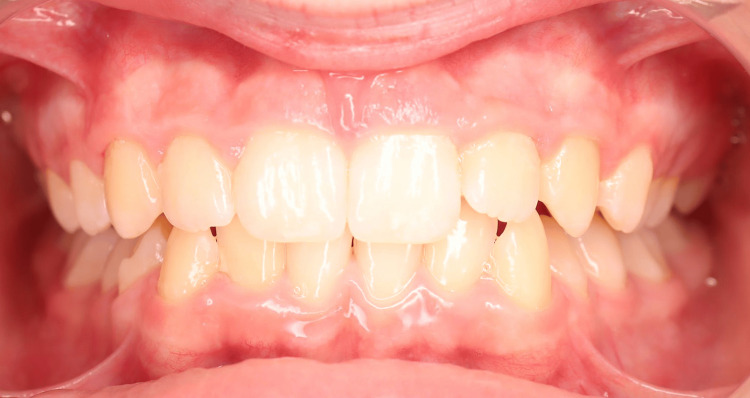
Intraoral photographs after wearing Myosimple for three months (frontal view).

**Figure 14:**
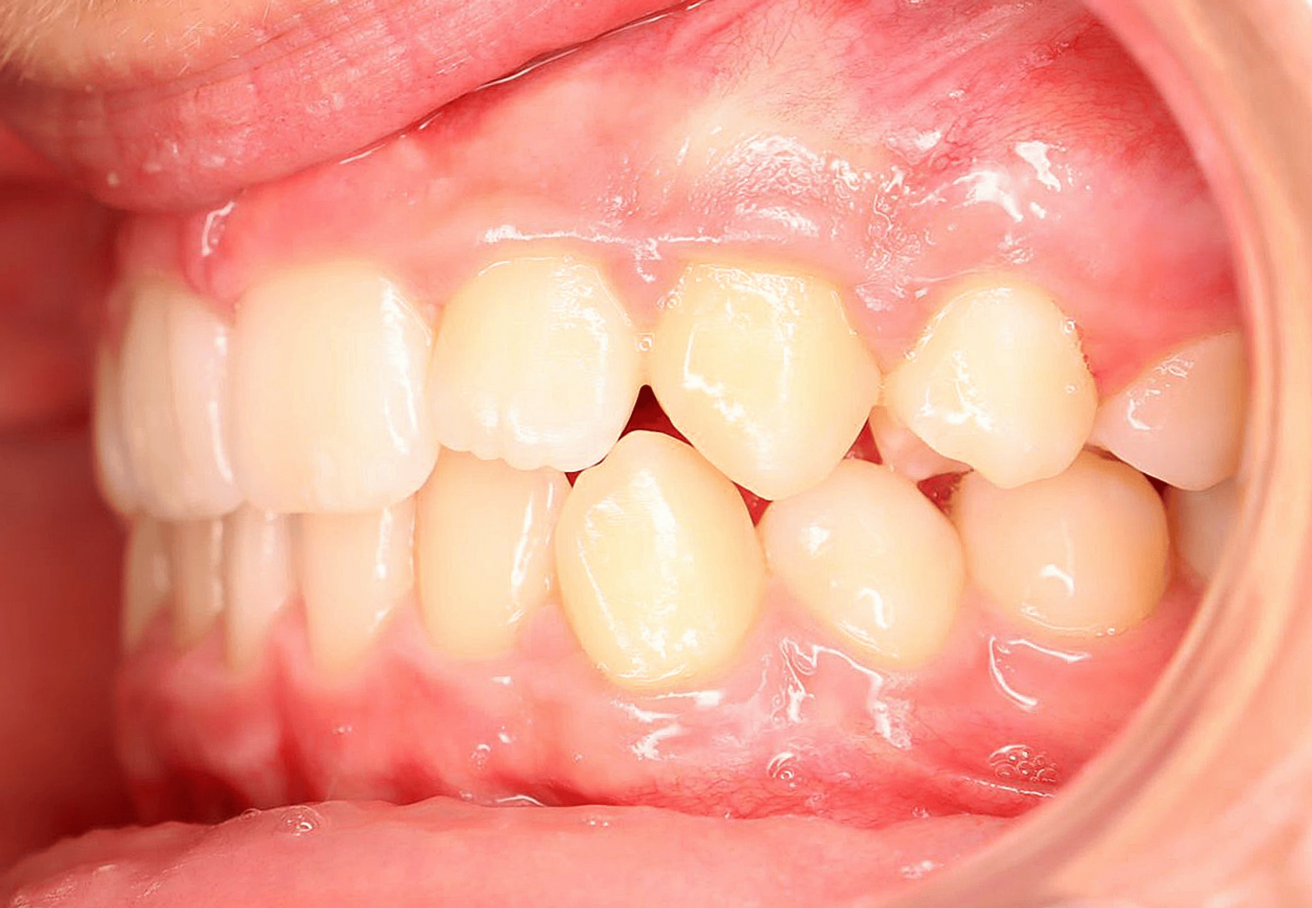
Intraoral photographs after wearing Myosimple for three months (left view).

**Figure 15:**
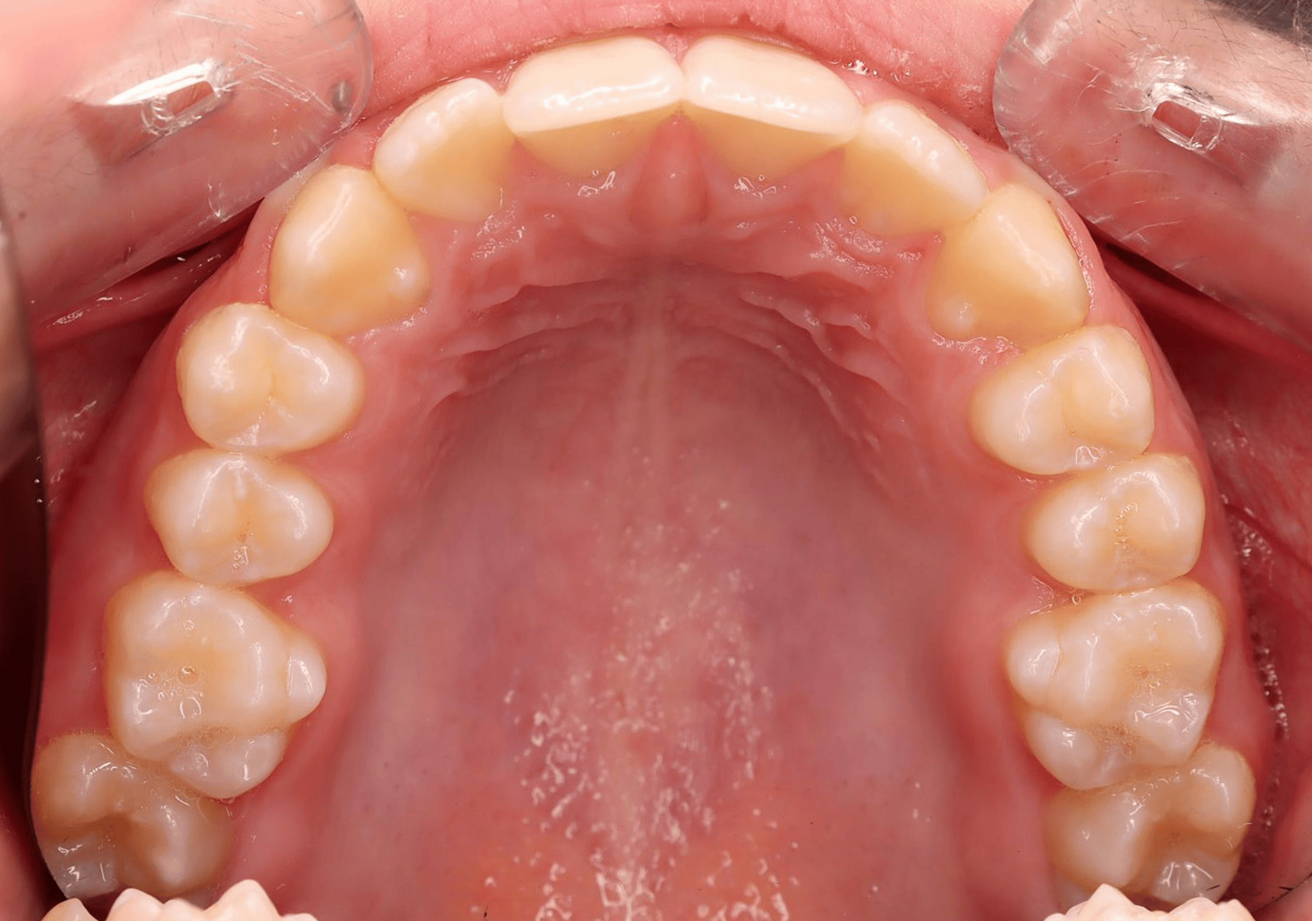
Intraoral photographs after wearing Myosimple for three months (maxilla occlusal view).

**Figure 16:**
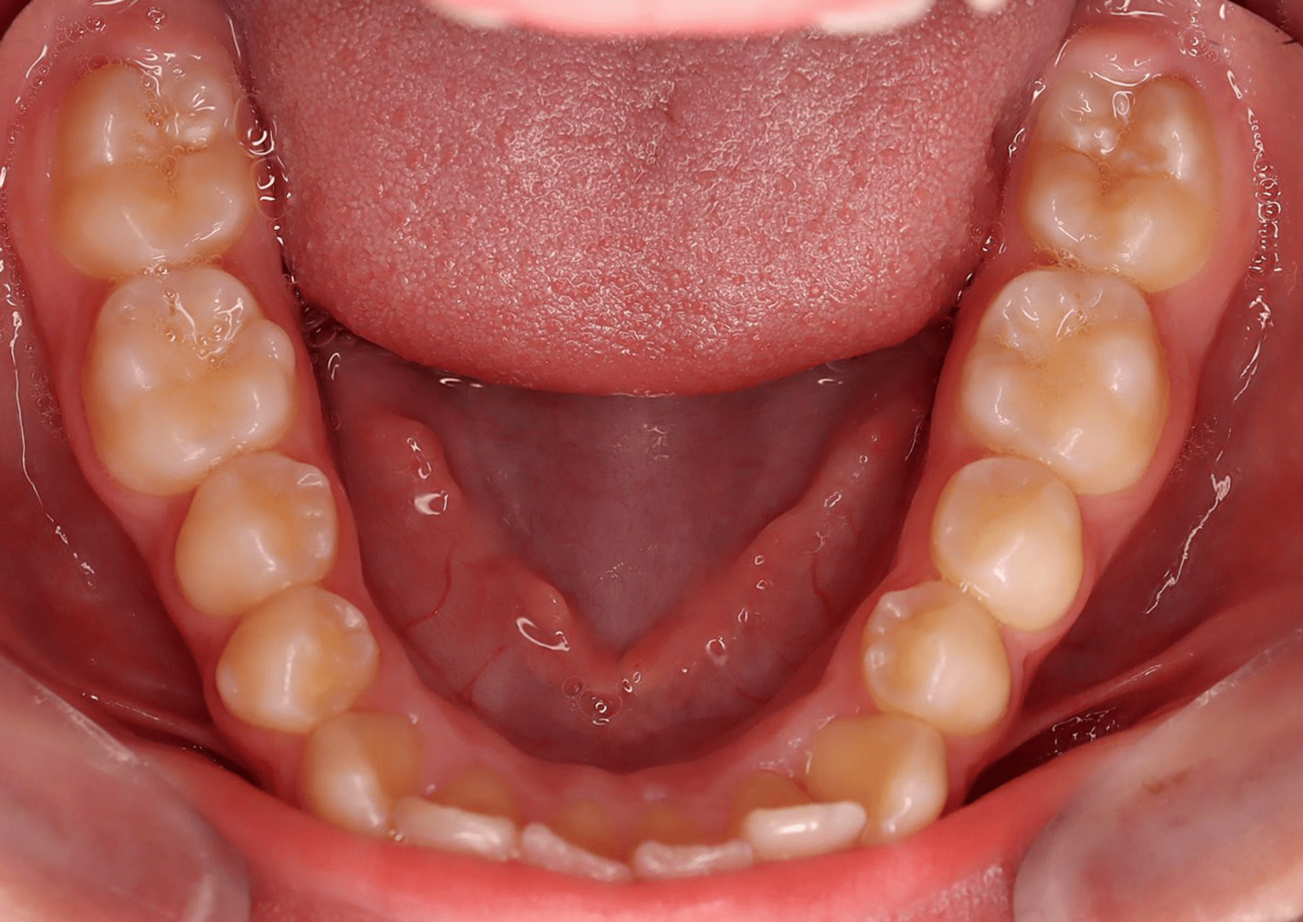
Intraoral photographs after wearing Myosimple for three months (mandibular occlusal view).

Wearing the Myosimple also resulted in better coordination of the dental arches and a reduction in maxillary and mandibular crowding (Figures 12-16). It also led to the acquisition of functional swallowing.

**Figure 17:**
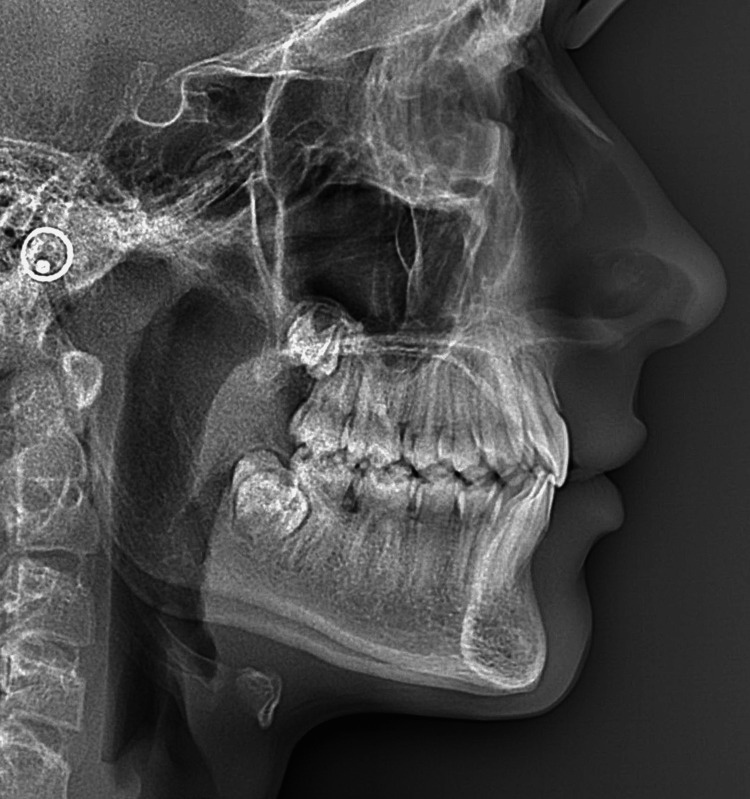
Profile teleradiograph after wearing Myosimple for three months.

After wearing Myosimple for three months, the profile teleradiograph shows the correction of the incisive overbite and of the retroclination of upper and lower incisors (Figure 17).

**Figure 18:**
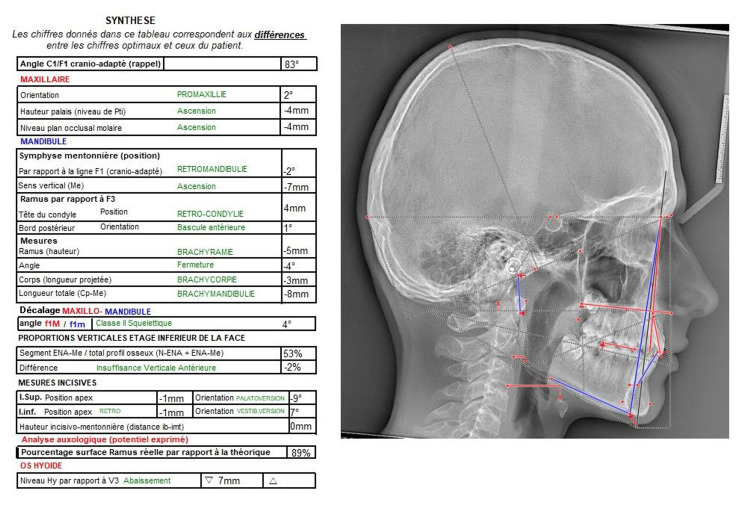
Prof. Jean Delaire's cephalometric analysis of the post-treatment profile teleradiograph.

Prof. Jean Delaire's cephalometric analysis of the post-treatment profile teleradiograph shows an improvement in the incisal axes. The initial lingual inclination of the maxillary central incisors of -24° was now only -9°. The initial lingual inclination of the mandibular incisors of -4° has been corrected to a vestibular inclination of +7° (Figure 18).

## Discussion

Prolonged orthodontic treatment exposes patients to potential iatrogenic effects such as enamel demineralization [8] and root resorption [9], as well as a risk of psychological fatigue and reduced treatment compliance [10]. Various approaches, both non-surgical [11] and surgical [12], have been proposed to accelerate orthodontic tooth movement. Among the various approaches proposed to accelerate orthodontic tooth movement, surgical procedures such as corticotomy or piezocision, photobiomodulation, pulsed electromagnetic fields, and the use of custom-made orthodontic appliances have been the most widely published .

In addition to accelerating orthodontic tooth movement, there is another approach that can reduce the duration of the phase using multi-attachments or aligners. This approach, described in this article, consists of starting treatment with a phase of OMT assisted by the wearing of a rigid PFA like the Myosimple.

Correction of an incisal overbite may require a removable retro-incisal plan, a fixed anterior disocclusion plate, temporary orthodontic anchors, nickel-titanium mandibular arches with an inverted Spee curve, bonded anterior bite blocks, anterior bite ramps integrated into the maxillary aligner or OMT with Myosimple.

Even if solid evidence has not yet been published, I believe it is advisable, except for surgical cases, to precede multi-attachment or aligner orthodontic treatment with an initial phase of OMT assisted by PFA, in order to achieve two therapeutic objectives.

The first benefit is to ensure the standard therapeutic management of orofacial dysfunction. Failure of OMT after three months of follow-up requires a simple explanation and may lead to suspension of treatment. If no initial OMT is undertaken and the persistence of orofacial dysfunctions disrupts the course of treatment and the achievement of its objectives, or worse, leads to relapse after treatment, the practitioner will be required not only to explain but also to justify his actions.

The second advantage is the improvement of occlusion. Wearing a rigid PFA generally allows significant overbite to be corrected within a few months, improves maxillary and mandibular crowding, and initiates distalization of the lateral sectors. Clinical experience shows that a rigid PFA is more effective in achieving this goal than soft PFAs. The improvement obtained in occlusion allows treatment to continue with a multi-attachment or aligner phase, which is then more effective and faster.

In the case of this patient, aged 11 years and 8 months, the initial phase of OMT assisted by the regular use of Myosimple for three months resulted in better coordination of the dental arches and a reduction in maxillary and mandibular crowding. It also led to the acquisition of functional swallowing. Wearing the Myosimple also resulted in an improvement in the incisal axes. The initial lingual inclination of the maxillary central incisors of -24° was now only -9°. The initial lingual inclination of the mandibular incisors of -4° has been corrected to a vestibular inclination of +7°. 

**Figure 19:**
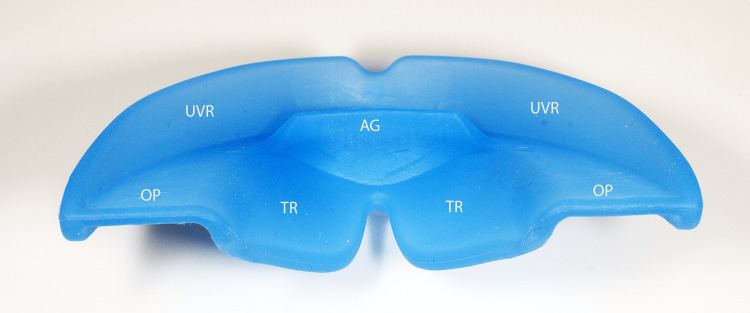
The Myosimple, a new innovative PFA (upper posterior-anterior view). PFA: prefabricated functional appliance; UVR: upper vestibular rim; LVR: lower vestibular rim; OP: occlusal plane; TR: tongue ramp; AG: anterior guide.

**Figure 20:**
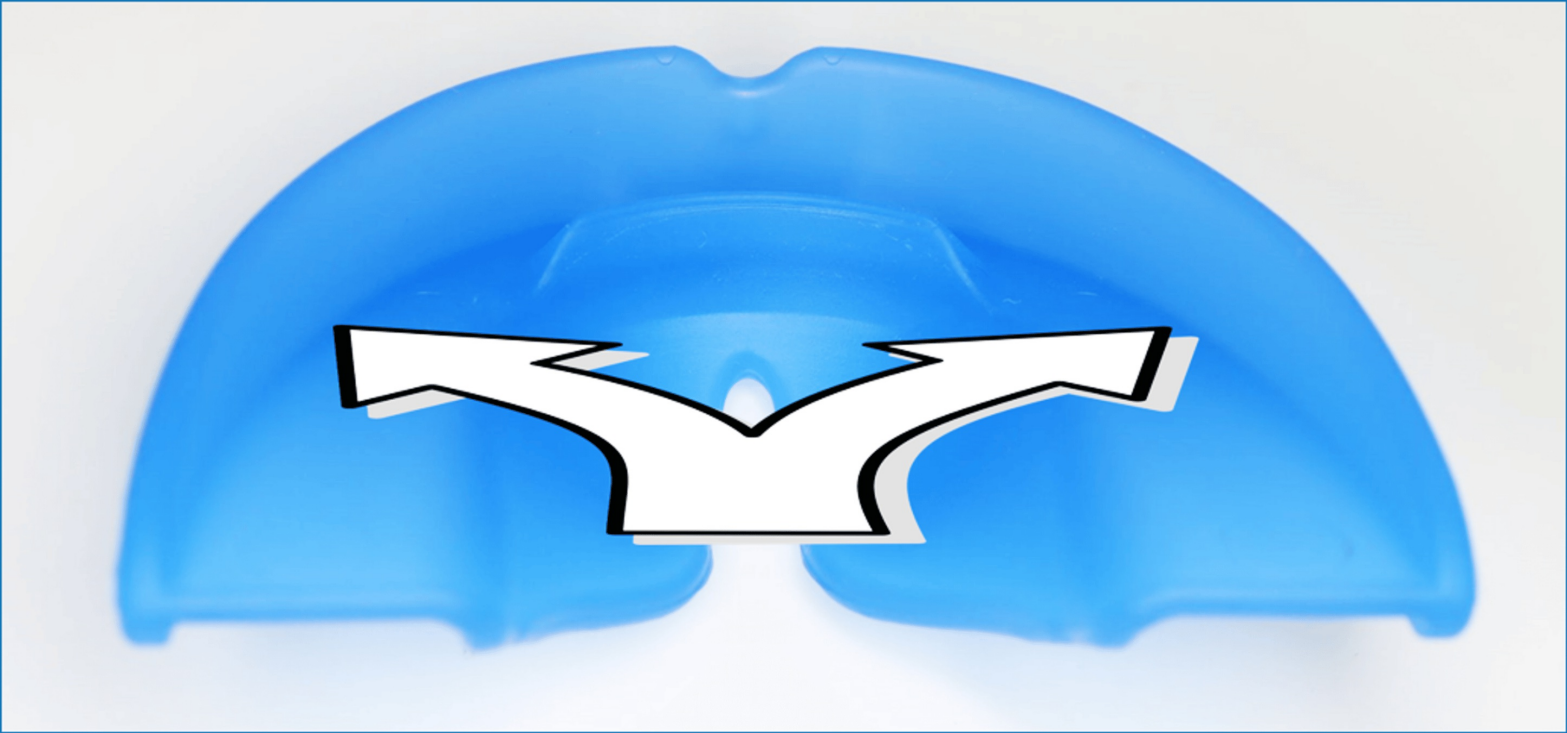
The Myosimple (tongue elevation and protrusion).

**Figure 21:**
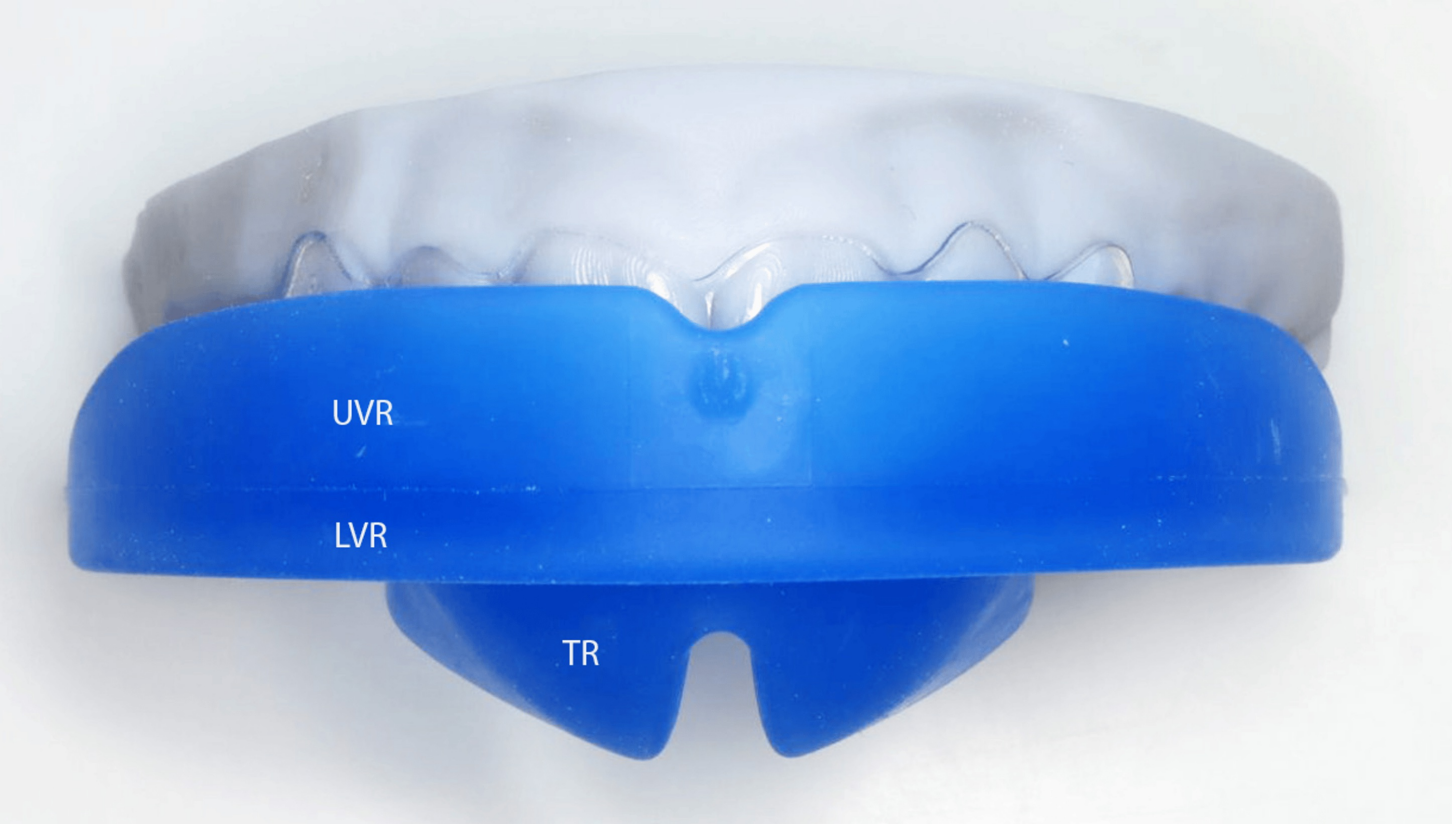
The Myosimple (frontal view). UVR: upper vestibular rim; LVR: lower vestibular rim; TR: tongue ramp.

It is the innovative and patented features of Myosimple [13] that enable the effective implementation of this OMT protocol with rigid PFA. I believe that Myosimple represents a significant improvement on existing PFA, combining effectiveness, safety, and ease of use. Its features, each of which serves one or more specific purposes, have already been described in detail [13], so I will only highlight the main ones here (Figures 19-21).

The material used for the PFA is rigid, medical grade with a medical colorant. It meets the main standards and does not contain phthalates, endocrine disruptors or bisphenol. Preference was given to a thermoplastic elastomer (TPE) with a hardness of approximately 80 Shore A and sterilizable at 134°C. The rigidity of the material helps prevent degradation of the PFA while maintaining excellent wearing comfort thanks to its very small size. This may seem counterintuitive, but it is immediately accepted by patients who feel more stable and comfortable compared to a soft PFA. The rigidity of the material helps to reshape the dental arches and lift excessive incisal coverage (Figures 2, 13).

The height of the upper vestibular rim (UVR) is reduced to prevent injury to the maxillary mucosa, especially at the canine bumps and on the inside of the cheeks and lips. This ensures good stability of the Myosimple in the mouth and imposes nasal ventilation after nasal permeability has been verified.

The reduced height of the lower vestibular rim (LVR) to 1.2 mm, sufficient to ensure mandibular positioning and support, allows muscle pressure to be exerted on the mandibular alveolar arch. This feature allows it to be worn in parallel with a multi-attachment device or aligners if additional OMT is required.

A specific TR induces passive elevation of the lingual dome thanks to the absence of the rim that usually borders the upper part of the occlusal plane (OP) of other PFA on the lingual side. The continuity between the TR and the OP is patented (Figures 14, 15). This combination allows the tongue to meet the entire palatal mucosa and the palatal surfaces of the maxillary premolar-molar sector. It promotes lingual propulsion and clears the upper airways [14].

## Conclusions

Achieving functional balance is a prerequisite for effective orthodontic treatment and stable results. This balance can be achieved more effectively through OMT when combined with the use of a PFA. A significant improvement in the shape and relationship of dental arches can be achieved by starting orthodontic treatment with a phase of OMT assisted by the regular use of Myosimple. This new PFA is rigid and immediately well accepted and worn thanks to its innovative design and very small size. As a result of the improved occlusion achieved, the subsequent treatment with a multi-attachment or aligner phase is more effective and faster.
